# Nomophobia Profiles Among High School and College Students: A Multi-Group Latent Profile Analysis

**DOI:** 10.3390/bs15091282

**Published:** 2025-09-18

**Authors:** Wenqin Chen, Bin Gao, Yang Zhou, Xiaoqi Yan

**Affiliations:** 1 School of Education, Shanghai Normal University, Shanghai 200234, China1000529935@smail.shnu.edu.cn (X.Y.); 2 College of Teacher Education, Hunan City University, Yiyang 413000, China; 3 School of Teacher Education, Hubei Minzu University, Enshi 445000, China

**Keywords:** nomophobia, latent profile analysis, high school students, college students

## Abstract

In school settings, nomophobia—a newly identified form of problematic mobile phone use characterized by anxiety and discomfort experienced when an individual is unable to use or access their smartphone—poses significant challenges to students’ learning and daily life. Prior research on nomophobia has predominantly adopted a variable-centered perspective. However, if nomophobia is heterogeneous across subgroups, acknowledging this heterogeneity may inform the advancement of more tailored and productive therapeutic methods. Latent profile analysis (LPA) was conducted separately among high school students (N = 446) and college students (N = 667) to identify potential subgroup heterogeneity in nomophobia. To examine cross-group similarities in nomophobia profiles, a multi-group LPA was employed. Based on multiple model fit criteria, a three-profile solution—high nomophobia, moderate nomophobia, and low nomophobia—was identified for both groups. However, the multi-group LPA provided only partial support for the similarity of nomophobia profiles across educational stages, specifically in terms of configural and dispersion similarity. While similar nomophobia profiles emerged across groups, the partial equivalence suggests that intervention strategies for nomophobia may not be universally applicable across different educational levels. Additional studies should investigate the mechanisms underlying students’ nomophobia profiles and to inform differentiated interventions for educators, institutions, and policymakers.

## 1. Introduction

With the continuous evolution and upgrading of mobile internet technologies, mobile phones are now deeply embedded in individuals’ daily activities, facilitating a wide range of activities such as online learning, social interaction, entertainment, and self-presentation through social networking sites and digital social platforms (Troussas et al., 2021; Hoffner & Bond, 2022; Nwagwu & Akintoye, 2023). For example, as recorded in December 2024, China’s mobile internet subscriber population had reached 1.105 billion, accounting for 99.7% of all internet users (CNNIC, 2025). Similarly, by the end of 2022, the internet penetration rate among minors in China had reached 97.2%, approaching full saturation. Approximately 90% of minors owned their own internet-enabled devices and primarily accessed the internet via mobile phones (CNNIC, 2023). This widespread reliance on mobile phones has raised concerns about potential psychological consequences, one of which is nomophobia—the anxiety and discomfort experienced when one is unable to use or does not have access to their smartphone (King et al., 2010, 2013). Nomophobia is prevalent across various contexts, including education, work, and daily life, and significantly impacts individuals’ cognition, behavior, and well-being. For example, in educational settings, increased nomophobia levels predict reduced academic success (Mengi et al., 2020; Durak, 2019). In the workplace, employees with higher nomophobia show reduced productivity (G. Wang & Suh, 2018). Similarly, drivers with elevated nomophobia are associated with risky phone-while-driving behaviors (Kaviani et al., 2020). Furthermore, higher nomophobia severity is associated with greater somatic symptoms, particularly dizziness and impaired sleep quality (Jilisha et al., 2019; Jahrami et al., 2024).

Given these wide-ranging consequences, scholars have increasingly examined not only the outcomes but also the underlying risk factors that contribute to the development and persistence of nomophobia. Previous variable-centered studies have identified a range of risk factors associated with nomophobia, such as smartphone checking frequency (Aslani et al., 2025), loneliness, and diminished impulse control (Arpaci, 2022), as well as protective factors, including cooperation (Olivencia-Carrión et al., 2018), basic psychological needs (Sezer & Yıldırım, 2020), and physical activity or associativism (Gonçalves et al., 2020). These studies typically assume that all individuals come from a single homogeneous population and that the sample reflects an underlying structure common across potential subgroups (Morin et al., 2016). However, this assumption may not always hold, as individuals’ smartphone use habits, contexts, and conditions can vary considerably across different groups.

### 1.1. Literature Review

Drawing on the functional perspective of mobile phones—such as portability and their ability to fulfill individualized psychological needs—researchers have proposed the self-expansion theory (Wu et al., 2022; Ross & Kushlev, 2025). According to this framework, individuals may engage in self-expansion through various functionalities of smartphones (Davel, 2017). For instance, smartphones’ capabilities in memory storage, computation, and illumination can extend one’s cognitive and physical capacities. Consequently, individuals may perceive their phones as an integral part of themselves, referring to them as an extension of the hand or a “brain in the pocket” (Park & Kaye, 2019). Research evidence indicates that habitual smartphone users for self-expansion tend to exhibit elevated levels of affective distress (e.g., social isolation, worry, melancholy, and ennui) and diminished positive emotions (e.g., happiness) when they are unable to access their phones (Hoffner et al., 2016; Mir & Akhtar, 2020).

Regarding the antecedents of nomophobia, demographic variables—including age, gender, and socioeconomic status (SES)—have been established as key determinants influencing its symptoms (Heng et al., 2023; Hessari et al., 2024). Specifically, research indicates that female students exhibit higher susceptibility to nomophobia than their male counterparts (Moreno-Guerrero et al., 2020). However, with respect to age, no significant differences have been observed among young individuals aged 12 to 20 years (Moreno-Guerrero et al., 2020). An integrative research analysis further highlights that females and young people appear to be particularly vulnerable to nomophobia (León-Mejía et al., 2021). Conversely, regional and residential factors, including geographical location and accommodation status, demonstrated no statistically meaningful correlation with nomophobia (El-Ashry et al., 2024). This suggests that static background characteristics, such as where individuals live or whether they reside on or off campus, may not independently explain variations in nomophobia. Instead, behavioral patterns appear more salient. For instance, average daily smartphone usage has been found to positively predict nomophobia (El-Ashry et al., 2024). While some studies have employed descriptive analyses to explore demographic differences in nomophobia (Arpaci, 2022), others have examined sociodemographic factors—such as gender—as control or moderating variables (Guimarães et al., 2022; Bulut & Sengul, 2023). Nevertheless, limited research has implemented a holistic perspective to analyze how these factors contribute to distinct profiles or subtypes of nomophobia. In addition to sociodemographic predictors, fear of missing out (FoMO) has emerged as critical risk factors for nomophobia (Wibowo & Safaria, 2025). For instance, FoMO has been shown to significantly and positively predict nomophobia symptoms among both high school and university students (Ergin & Ozer, 2023; Wibowo & Safaria, 2025).

Although nomophobia is a multifaceted construct (Yildirim & Correia, 2015), most existing research has relied on composite scores to assess it, potentially overlooking meaningful variation across individuals. In fact, diverse subtypes of nomophobia symptoms can be identified through person-centered methods. Latent profile analysis (LPA) has been employed in studies of nomophobia to delineate different symptom profiles across various populations. These studies frequently find that nomophobia profiles are mainly categorized by symptom severity. For instance, Luo et al. (2024) used LPA to identify three nomophobia classes among college students, and as such were labeled “no-risk nomophobia”, “low-risk nomophobia”, and “at-risk nomophobia”. Similarly, Liu et al. (2024) conducted a study on 933 nursing students in China and identified three latent classes of nomophobia: mild nomophobia (10.5%), moderate nomophobia (53.6%), and severe nomophobia (35.9%). These findings align with the results of Rodríguez-Sabiote et al. (2020), who proposed that three distinct groups—high, medium, and low—best represent nomophobia symptoms, underscoring the importance of a nuanced understanding of this phenomenon. Further supporting this perspective, El-Ashry et al. (2024) reported that the majority of individuals experience varying degrees of nomophobia, with 40.3% classified as having severe symptoms, indicating a strong dependence on mobile phones. Moderate and mild nomophobia were observed in 28.5% and 27.5% of participants, respectively, while only 3.6% showed no symptoms. Adding to this body of evidence, a random-effects meta-analysis by Jahrami et al. (2023) revealed that roughly 20% of individuals experience mild nomophobia, about half report moderate symptoms, and another 20% present with severe symptoms. Together, these studies highlight the diverse nature of nomophobia symptom profiles and emphasize the significance of identifying distinct subtypes to improve the effectiveness of tailored interventions.

Furthermore, there is limited knowledge regarding the extent to which nomophobia profiles can be applied to diverse educational settings and demographic groups (Luo et al., 2024). To evaluate comprehensively how consistently latent profiles emerge across multiple samples, we adopted a profile similarity method to investigate both college and high school students (Morin et al., 2016). From a life course perspective, the shift from secondary to postsecondary education is a period during which school-age children’s mobile phone use behavior changes accordingly (Lepp et al., 2014). Studies of student populations with different environmental contexts (e.g., college vs. high school) can help to understand the effects of such transitions on individual mobile use behaviors and habits.

Despite the extensive body of research on nomophobia, several limitations emerge when evaluating past findings. First, most studies have taken a variable-centered approach, examining the links between nomophobia and its antecedents or outcomes within specific populations (Berdida & Grande, 2023; Aydin & Kuş, 2023). This approach may overlook key differences among subgroups within the overall sample. As a result, it can contribute to inconsistencies in findings across studies. Additionally, interventions designed to reduce nomophobia may be less effective, as they often treat all individuals similarly, without accounting for subgroup or individual variations. Second, most prior studies on nomophobia, including its determinants and outcomes, have focused on a single population or educational stage, particularly college students. These results might not be broadly applicable to alternative age cohorts or allow for comparisons of nomophobia across various educational levels. It is necessary to explore nomophobia across different age groups or educational stages to enable meaningful comparisons and better understand its variations.

#### The Study Context

China provides a distinctive educational context for investigating nomophobia, as students at different educational stages face markedly divergent academic and institutional environments. In secondary education, particularly at the high school level, students encounter considerable academic pressures due to the highly competitive National College Entrance Examination (Gaokao), which serves as the primary pathway for university admission (Zhao et al., 2015). This exam-oriented system requires extensive preparation, resulting in heavy workloads and intense study schedules for high school students (Gao et al., 2025). In contrast, college students in China experience a relatively more flexible and autonomous learning environment (Ding, 2017; J. Wang et al., 2024) where academic stress is reduced and extracurricular activities and social networking become more salient aspects of daily life. Institutional regulations further differentiate these two groups. The Ministry of Education of China (2021) has introduced policies that restrict primary and secondary school students from bringing mobile phones into school unless strictly necessary and explicitly prohibits their use in classrooms. These restrictions are intended to minimize distraction, promote learning efficiency, and reduce excessive reliance on mobile devices during school hours (Campbell et al., 2024). Such measures may buffer high school students from developing habitual smartphone dependency, potentially lowering their risk of experiencing severe nomophobia symptoms. By contrast, university students are not subject to comparable restrictions, enabling unrestricted access to mobile phones both inside and outside the classroom (Gao et al., 2021). This policy gap may inadvertently reinforce patterns of mobile phone overuse and exacerbate vulnerability to high-risk nomophobia profiles among college students. Together, these contextual distinctions—academic workload, institutional regulations, and levels of autonomy—underscore the importance of examining nomophobia across different educational stages in China.

### 1.2. Research Questions and Hypothesis Development

To date, few studies have examined whether nomophobia profile patterns emerge and remain stable across varying age cohorts or educational levels. This study therefore addresses three key questions: (a) Can nomophobia profiles be replicated in independent samples of high school and college students? (b) What demographic and individual factors predict profile membership, and how does social media use differ across profiles? (c) To what extent are the profiles comparable between high school and college students?

The primary objective was to identify nomophobia profiles among high school and college students separately. Building on prior research that revealed distinct subgroups of college students with varying levels of nomophobia symptoms (Luo et al., 2024), we expected the latent profiles to primarily differ in symptom severity. The second objective was to examine the degree of similarity between the two groups in terms of the number, structure, and distribution of profiles. We hypothesized that comparable profiles would emerge in both cohorts, showing a substantial degree of cross-group similarity. However, considering the transitional phase from high school to college and the varying smartphone usage regulations enforced by educational authorities in China (Ministry of Education of China, 2021), we hypothesized potential differences in profile means, variances, and the relative proportions of the nomophobia profile groups. The third objective was to identify the determinants of nomophobia symptom profiles by assessing how individual characteristics influence the probability of being classified into specific profiles. Building on prior studies that identified factors such as gender, age, place of residence, daily smartphone usage duration, and FoMO as significant predictors of nomophobia symptoms (León-Mejía et al., 2021; Ergin & Ozer, 2023; Kara et al., 2019), we posited that these individual traits and FoMO would serve as predictors of the various nomophobia subtypes. The fourth goal was to evaluate the connections between nomophobia profiles and their anticipated consequences of internet usage behavior. Drawing on earlier studies emphasizing the close connection between nomophobia; social networking site (SNS) usage, including WeChat, Weibo, and QQ; and social media usage (Bhattathirippad & Patel, 2021; Berdida & Grande, 2023), we hypothesized that profiles characterized by more severe nomophobia symptoms would be associated with higher levels of both active and passive SNS use.

**Figure 1:**
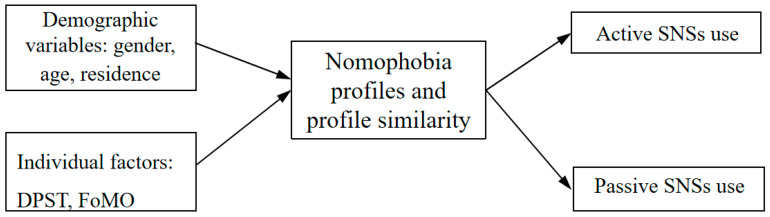
Conceptual framework in this study. Note. DPST = daily phone screen time, FoMO = fear of missing out, social networking site = SNS.

A conceptual framework was developed (see Figure 1) to depict the relationships investigated in this study, illustrating the role of demographic variables and individual factors that predict nomophobia profiles, which are then analyzed for their links with active and passive SNS use.

## 2. Materials and Methods

### 2.1. Participants and Procedure

Participants were 446 high school students and 667 college students from China. Table 1 presents the descriptive statistics for high school (N = 446) and college students (N = 667) across several key variables. Among high school students, 60.3% were female, with a mean age of 16.18 years (SD = 1.15), and 33.4% resided in urban areas. On average, they reported 3.88 h of smartphone use per week. For college students, 69.4% were female, with a mean age of 19.84 years (SD = 1.46), and 32.7% resided in urban areas. Their reported smartphone use averaged 6.70 h per day.

Participants were recruited via convenience sampling from multiple schools. Initially, school administrators were contacted and provided with information about the study, including its purpose, procedures, and confidentiality assurances. With administrative approval, mental health teachers at each school informed eligible students about the study during class meetings and invited them to participate voluntarily. Students who agreed were provided with a link or QR code to the Wenjuanxing online survey platform, which they could access using their smartphones or computers. Prior to participation, consent for participation was formally obtained from the students and their respective legal guardians. Participants could withdraw from the survey without penalty at any time, and no financial or material inducements were offered. Ethical approval for this study was granted by the ethics committee of the first author’s affiliated institution.

### 2.2. Measures

#### 2.2.1. The Nomophobia Questionnaire (NMP-Q)

The Chinese adaptation of the NMP-Q, developed by Ren et al. (2020), was used to measure nomophobia. The original NMP-Q was created by Yildirim and Correia (2015). This revised version contains 16 items that evaluate the same four dimensions as the original—fear of losing contact, fear of losing internet connection, fear of not having access to information, and fear of losing convenience—and has demonstrated strong reliability (Ren et al., 2020). Responses are scored on a seven-point Likert scale, with options ranging from 1 (strongly disagree) to 7 (strongly agree). In this study, Cronbach’s α was 0.95 for high school students and 0.94 for college students.

#### 2.2.2. Fear of Missing out Scale

We measured FoMO using the eight-item Chinese version of the scale (Li et al., 2019), which was originally developed by Przybylski et al. (2013). Each statement was evaluated using a five-point Likert scale ranging from 1 (not at all true of me) to 5 (extremely true of me), with higher scores reflecting greater levels of FoMO. In this study, Cronbach’s alpha for high school students was 0.83 and 0.76 for college students.

#### 2.2.3. The Passive SNS Use Questionnaire

The Passive SNS Use Questionnaire, originally developed by Tandoc et al. (2015), was used to measure passive social networking site (SNS) usage. This single-factor scale comprises four items, such as “Browsing aggregated news feeds on social networking sites.” Participants rated each item on a 5-point Likert scale ranging from 1 (never) to 5 (frequently). Elevated total scores correspond to increased passive SNS usage.

#### 2.2.4. The Active SNS Use Questionnaire

The active use of SNSs among participants was assessed using the Active SNS Use Questionnaire developed by Frison and Eggermont (2015). This questionnaire primarily measures the frequency of active behaviors on SNSs in daily life, such as updating information, posting photos, and sharing images. It comprises five items, including examples like: “Please evaluate the frequency with which you chat with someone on social networking sites based on your actual usage.” All items are rated on a 5-point Likert scale ranging from 1 to 5. Total scores, derived from the sum of all items, indicate the frequency of active SNS usage, with higher scores representing greater engagement.

### 2.3. Statistical Analyses

Descriptive analyses and scale reliability were performed using SPSS 27.0, while the measurement invariance across gender and educational stage, as well as latent profile analyses (LPAs), were conducted in Mplus 8.3, utilizing the robust maximum likelihood (MLR) estimator (Muthén & Muthén, 1998–2017). Prior to the LPA, the data were examined for normality and were found to meet the assumptions for subsequent analyses. Various statistical tests and indices were employed to determine the optimal number of profiles in the LPA. Additionally, entropy, which measures how accurately cases are assigned to profiles, was assessed. The entropy statistic, bounded between 0 and 1, measures classification reliability, whereby larger values represent enhanced accuracy (Nylund-Gibson & Choi, 2018).

According to established guidelines, fit indices such as TLI and CFI exceeding 0.90 are considered acceptable, with values surpassing 0.95 reflecting excellent model fit (Hu & Bentler, 1999). Based on these criteria, we proceeded to evaluate measurement invariance by adopting a stepwise testing approach, which included assessments of configural, metric, and scalar invariance (Putnick & Bornstein, 2016). Evidence for measurement invariance is indicated by a non-significant chi-square difference between the unconstrained and constrained models. However, because chi-square values are easily influenced by sample size, changes in CFI (ΔCFI) are also evaluated to compare nested models. A ΔCFI below 0.01 indicates clear invariance, while values between 0.01 and 0.02 suggest probable invariance. A ΔCFI above 0.02 implies a lack of invariance. Furthermore, a ΔRMSEA exceeding 0.015 indicates that measurement invariance is not supported (Cheung & Rensvold, 2002; Chen, 2007).

To examine the similarity of nomophobia profiles across age groups, a series of increasingly restrictive multi-group comparisons were conducted following the procedures outlined by Morin et al. (2016), including assessments of configural, structural, dispersion, and distributional similarity. Based on the optimal profile enumeration model identified in the previous step, multi-group latent profile analyses (LPAs) were estimated to test (a) configural similarity (i.e., identical model structure across groups), (b) structural similarity (i.e., equality of profile-specific means), (c) dispersion similarity (i.e., equality of variances), and (d) distributional similarity (i.e., equality of profile membership proportions). These models were used to determine the extent to which the identified nomophobia profiles were comparable across age groups. Finally, multinomial logistic regression and chi-square tests were employed to examine the predictors and outcome differences associated with profile membership, respectively.

## 3. Results

### 3.1. Preliminary Analyses

Table 2 presents the differences in nomophobia and its four dimensions between the two groups. The results indicate significant differences across all variables. College students reported more pronounced nomophobia symptoms relative to high school students, with a small effect size (Cohen’s d = 0.27). For the dimension of not being able to access information, college students scored higher, indicating a small effect size (Cohen’s d = 0.18). In the dimension of giving up convenience, college students again scored higher, suggesting a small effect size (Cohen’s d = 0.16). Regarding losing connectedness, college students reported higher levels, indicating a small to medium effect size (Cohen’s d = 0.29). Lastly, for the dimension of not being able to communicate, college students scored higher, indicating a small to medium effect size (Cohen’s d = 0.30). These findings suggest that college students experience higher levels of nomophobia and its dimensions compared to high school students, with small to medium effect sizes indicating the magnitude of these differences.

### 3.2. Measurement Invariance Tests

Measurement invariance of the NMP-Q across gender and educational stages was tested separately. First, gender-based measurement invariance was assessed. The baseline configural model demonstrated a good fit (CFI = 0.930, TLI = 0.914, SRMR = 0.046, RMSEA = 0.070). Metric invariance was confirmed (ΔCFI = −0.001, ΔRMSEA = −0.002), and scalar invariance was also established (ΔCFI = −0.004, ΔRMSEA = 0.000) (see Table 3). Next, measurement invariance across educational stages was examined. The configural model showed a good fit (CFI = 0.924, TLI = 0.908, SRMR = 0.047, RMSEA = 0.073). Metric invariance was confirmed (ΔCFI = −0.006, ΔRMSEA = 0.000), while scalar invariance was not fully established (ΔCFI = −0.017, ΔRMSEA = 0.006). However, partial scalar invariance, allowing for non-equal intercepts on items 10, 11, 12, 14, 15, and 16, was supported (ΔCFI = −0.008, ΔRMSEA = 0.003).

### 3.3. Multi-Group Latent Profile Analysis

**Figure 2:**
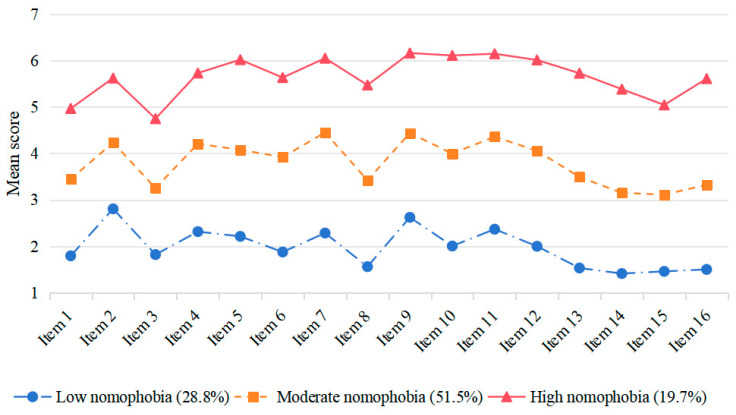
The three-profile solution of nomophobia for high school students.

**Figure 3:**
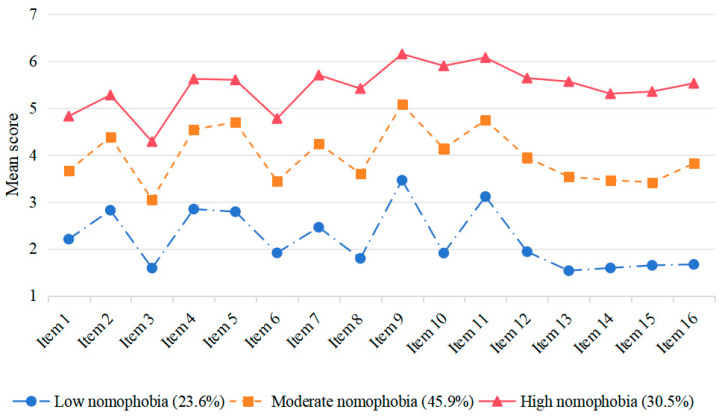
The three-profile solution of nomophobia for college students.

We conducted LPA to assess two- to four-profile solutions of nomophobia for both high school and college students. Table 4 presents the respective fit indices for the profile solutions for the two groups. The mean scores of each item in the two groups are shown separately in Figure 2 and Figure 3. For high school students, the three-profile solution showed the best balance between model fit and parsimony, with high classification accuracy (entropy = 0.951) and significant improvements over the one- and two-profile solutions. Although the four-profile solution had slightly better fit indices, it did not significantly improve the model according to the LMRT *p*-value. Similarly, for college students, the three-profile solution provided the optimal balance with high classification accuracy (entropy = 0.917) and significant improvements indicated by the BLRT and LMRT *p*-values. The four-profile solution, despite having the lowest AIC and BIC values, did not show a significant improvement over the three-profile solution. These findings suggest that the three-profile solution (low nomophobia, moderate nomophobia, and high nomophobia) is the most appropriate for both high school and college students, providing a meaningful differentiation of nomophobia levels.

### 3.4. Multi-Group Profile Similarity Analysis

Table 5 displays the model fit indices for the multigroup LPA across the two groups. First, to assess whether an identical number of profiles emerged across samples (i.e., configural similarity), we estimated a three-profile configural model across educational stages with all parameters freely varying. This baseline model served as the foundation for subsequent analyses. Fit indices supported retaining the three-profile solution for both samples, thereby confirming configural similarity across educational stages. Second, we tested structural similarity by constraining the within-profile means to be equal across educational stages. Fit indices for this structural model were compared with those of the configural model, in which means were freely estimated. The structural model yielded higher AIC, BIC, and aBIC values, indicating that structural similarity—identical within-profile means—was not supported between high school and college students. Third, dispersion similarity was tested by constraining the variances in the structural similarity model to be equal, assessing whether the indicators’ variability was consistent across educational stages. The model fit showed slight decreases in AIC, BIC, and aBIC, indicating that dispersion similarity (i.e., equal within-profile variances) was acceptable across educational stages. Finally, distributional similarity was assessed by imposing equality constraints on the relative size of the profiles (i.e., class probabilities) across educational stages. The resulting increases in AIC, BIC, and aBIC values indicated that distributional similarity was not supported. Consequently, we concluded that the relative profile sizes were not consistent between high school and college students.

### 3.5. Nomophobia Profile Predictors

Multinomial logistic regression outcomes are shown in Table 6, evaluating the probability of belonging to high or moderate nomophobia profiles versus the low profile. Odds ratios (OR) and regression coefficients (B) are reported for gender, age, residence, daily phone screen time (DPST), and FoMO.

For college students, neither gender, age, nor residence significantly predicted membership in the high or moderate nomophobia profiles. Similarly, DPST did not emerge as a significant predictor. However, FoMO was a highly significant predictor: students with higher levels of FoMO were 12.60 times more likely to belong to the high nomophobia profile (B = 2.53, OR = 12.60, *p* < 0.001) and 4.61 times more likely to be in the moderate profile (B = 1.53, OR = 4.61, *p* < 0.001).

For high school students, gender and residence were not significant predictors for either nomophobia profile. However, age was a significant predictor for the high nomophobia profile, with younger students being 33% less likely to be in the high profile compared to the low profile (B = −0.39, OR = 0.67, *p* < 0.01), though age was not a significant predictor for the moderate profile. DPST was a significant predictor for both the high and moderate profiles, with students who spent more time on their phones being 1.15 times more likely to be in the high profile (B = 0.14, OR = 1.15, *p* < 0.001) and 1.12 times more likely to be in the moderate profile (B = 0.11, OR = 1.12, *p* < 0.01). As with college students, FoMO was the strongest predictor, with high FoMO students being 13.92 times more likely to be in the high nomophobia profile (B = 2.63, OR = 13.92, *p* < 0.001) and 3.68 times more likely to be in the moderate profile (B = 1.30, OR = 3.68, *p* < 0.001). These results underscore FoMO as the most powerful predictor of nomophobia across both educational levels, with age and DPST also being significant factors among high school students. Gender and residence were not significant predictors for either group.

### 3.6. Nomophobia Profile Differences in Active and Passive SNS Use

Table 7 presents significant differences in both active and passive SNS use across the high, moderate, and low nomophobia profiles among both college and high school students. Generally, individuals classified in the high nomophobia profile reported higher levels of engagement with SNSs—both active and passive—compared to those in the moderate and low profiles. These differences were statistically significant across groups. Effect sizes indicated small-to-moderate differences among college students and moderate-to-large differences among high school students, suggesting that the association between nomophobia and SNS use is more pronounced during adolescence. The observed patterns highlight a positive gradient between the severity of nomophobia and the frequency of SNS engagement.

## 4. Discussion

The present study examined various profiles of nomophobia using four key indicators: the inability to access information, loss of convenience, loss of connectedness, and the inability to communicate. The findings extend the current body of research on nomophobia. Specifically, three distinct profiles emerged, ranging from low to high risk of nomophobia. In line with the study by Liu et al. (2024), which identified three nomophobia profiles—mild, moderate, and severe—among Chinese college students, the present study also revealed three latent subgroups with distinctive combinations of nomophobia indicators. This aligns with previous research (Luo et al., 2024; Rodríguez-Sabiote et al., 2020), which also found similar subgroups using LPA. The first profile consisted of regular mobile phone users, who scored low on the nomophobia scale. The second profile included moderate mobile phone users, who exhibited a moderate risk of developing nomophobia. Notably, this group, while showing a preference for mobile phone use, demonstrated only a moderate level of nomophobia, which aligns with prior research suggesting that mobile phone dependency does not always translate into problematic or addictive behavior (Kuss & Griffiths, 2017). The third profile was made up of individuals with a high level of mobile phone dependency, who exhibited high levels of nomophobia. From a theoretical perspective, these findings reinforce the view that nomophobia represents a heterogeneous construct rather than a homogeneous syndrome. The identification of three distinct subgroups supports dimensional models of technology-related anxieties, which emphasize variability in symptom severity and underlying psychological mechanisms. Furthermore, the emergence of consistent profiles across independent samples suggests that the structure of nomophobia may be relatively stable and generalizable, lending support to the theoretical argument that nomophobia reflects broader processes of digital dependency and psychological need for connectivity. Finally, the differentiation between moderate and high-risk groups highlights the importance of conceptualizing nomophobia not merely as a binary condition (present vs. absent) but as a spectrum, which allows for more nuanced theorizing about its antecedents and consequences.

An additional goal was to investigate the generalizability of nomophobia profile structures across secondary and postsecondary student groups. The results supported our hypothesis that, although both samples yielded an identical number of latent nomophobia profiles, there were nuanced differences within these profiles. Specifically, the number of nomophobia profiles was consistent across the two samples, suggesting that the three-profile solution is applicable for latent profiling of students across both educational stages. Additionally, dispersion similarity (i.e., equal within-profile variances) was found to be acceptable across these two groups. However, structural similarity (i.e., identical within-profile means) was not supported between the samples. This finding aligns with the *t*-test results of the present study, which showed that college students experience higher levels of nomophobia and its dimensions compared to high school students. The effect sizes were small to medium, indicating the magnitude of these differences (Leppink et al., 2016). Furthermore, distributional similarity (i.e., class probabilities) was not supported, meaning the distribution of students across the three profiles varied slightly between the two samples. Specifically, a larger proportion of college students were classified into the high-risk nomophobia profile. This difference is consistent with existing literature, which indicates that problematic mobile phone use is more frequent and severe among college students. For instance, a recent study revealed that Chinese adolescents spend an average of over 90 min per day on short video apps, while college students spend close to 180 min daily (Sun & Jiang, 2025).

Furthermore, the present study explored the antecedents of nomophobia profiles in both high school and college student samples. Consistent with previous findings, age, daily phone screen time, and FoMO emerged as significant predictors of nomophobia profiles (Hessari et al., 2024; Ergin & Ozer, 2023; Wibowo & Safaria, 2025). Specifically, the study revealed that lower-grade high school students were significantly more likely to fall into the high-risk nomophobia profile compared to upper-grade high school students. Additionally, as daily phone screen time increased (Fendel et al., 2025), high school students were more likely to be categorized into high-risk and moderate-risk nomophobia subgroups. FoMO was found to be a significant risk predictor for both high school and college students, increasing the likelihood of falling into high-risk and moderate-risk nomophobia subgroups. Theoretically, these findings underscore that nomophobia is not merely a behavioral outcome of phone use but is closely tied to developmental and psychological mechanisms. The higher risk among younger adolescents reflects the immaturity of self-regulation capacities, consistent with developmental models of impulse control. The role of screen time supports reinforcement-based theories, in which habitual device engagement strengthens dependency pathways. Meanwhile, FoMO aligns with self-determination theory (Lemay et al., 2019), suggesting that unmet needs for relatedness amplify reliance on mobile connectivity.

The chi-square analysis revealed significant differences in both active and passive social media use across the three identified nomophobia profiles. Generally, elevated nomophobia levels were linked to increased social media use (Berdida & Grande, 2023), with this effect being more pronounced among high school students. These results align with prior studies indicating that more severe nomophobia symptoms are correlated with increased social media use and problematic mobile phone behavior (Ayar et al., 2018; Berdida & Grande, 2023; Aydin & Kuş, 2023). These results underscore the importance of considering specific nomophobia symptom patterns when addressing the mental health issues of students. Theoretically, the stronger link between nomophobia and social media use supports compensatory internet use theory (Kardefelt-Winther, 2014), suggesting that students rely on online platforms to satisfy unmet social needs. The heightened effect among high school students aligns with socio-developmental perspectives emphasizing adolescence as a critical stage for peer approval and identity formation.

### 4.1. Practical Implications

The identification of distinct nomophobia profiles emphasizes the importance of adopting person-centered approaches when addressing psychological health issues. By categorizing individuals into different nomophobia profiles, this study highlights the need for more comprehensive tools beyond basic nomophobia scores. These tools can assist educators and counselors in developing targeted interventions for students based on their specific nomophobia patterns. Furthermore, the study examined profile similarity across high school and college student groups, revealing important distinctions that can inform tailored interventions at various educational stages.

Key predictors of higher-risk nomophobia profiles included lower-grade high school students, increased daily phone screen time, and higher levels of FoMO. These findings suggest the need for targeted prevention programs and intervention strategies for these high-risk groups. For instance, integrating mental health education into the orientation programs for students could help address nomophobia and FoMO. Encouraging students to participate in extracurricular activities—such as sports, fitness programs, or volunteer work—could also reduce unnecessary phone use, thereby lowering the likelihood of students falling into high-risk nomophobia profiles.

The present study also found that individuals at higher risk for nomophobia tend to engage in both active and passive social media use. From an intervention perspective, it is critical to address the negative impact of nomophobia on students’ academic and personal lives. Assessing and implementing effective intervention strategies to reduce nomophobia is therefore of significant practical importance. Research suggests that several approaches have proven effective in treating nomophobia, including mindfulness-based therapies, cognitive behavioral therapy, mental health education, and existential therapy (Arpaci, 2017; Bragazzi & Del Puente, 2014). Additionally, the findings highlight the broader conceptual link between human experience and technology, suggesting that nomophobia can be understood not only as behavioral dependence but also as reflecting the intertwined relationship between individuals and their digital environment. This perspective enriches theoretical considerations of nomophobia across developmental stages.

### 4.2. Limitations and Future Directions of Research

The research is limited by its use of cross-sectional data. Much of the existing research on nomophobia primarily employs cross-sectional designs, with a notable absence of longitudinal studies (Rodríguez-García et al., 2020). A longitudinal approach would provide valuable insights into how nomophobia evolves and interacts with other variables over time. We believe that it would be particularly beneficial to examine how students transition from one educational stage to another using latent transition analysis or growth mixture modeling. These techniques could help track the trajectories of each latent profile over time, offering a more nuanced understanding of how nomophobia develops in response to potential interventions or as a result of individual growth through school transitions. Longitudinal models may also mitigate issues related to generalizability, particularly by utilizing two distinct cohorts, as was done in the present study. Additionally, we recognize that variations in sample sizes between the high school and college cohorts might have affected the accuracy of profile estimation. Cultural factors play a significant role in shaping nomophobia levels (Arpaci, 2017), and cross-national investigations have demonstrated considerable disparities in the intensity of nomophobia among university students from different countries (Gutiérrez-Puertas et al., 2019; Ozdemir et al., 2018). Future work could further investigate these cultural variations by conducting cross-cultural comparative studies to explore the similarities and contrasts in nomophobia across various cultural contexts. Furthermore, this study employed convenience sampling, which, while practical and commonly used in psychological research, may limit the representativeness of the sample. Future studies should consider probability-based or more diverse sampling strategies to enhance the generalizability of the findings. Finally, self-reported data may be subject to social desirability and recall biases, particularly regarding passive SNS use, as individuals may underreport socially undesirable behaviors or inaccurately estimate the amount of time spent on such activities. Future studies could address this limitation by incorporating objective behavioral measures (e.g., digital trace data or usage logs) and adopting experience sampling methods to capture real-time engagement, thereby improving the accuracy and ecological validity of assessments.

## 5. Conclusions

This study identified three distinct nomophobia profiles—low, moderate, and high risk—among high school and college students, providing empirical support for a person-centered approach to understanding nomophobia. Despite profile similarity across the two educational stages, nuanced differences emerged in the structure and distribution of these profiles, with college students showing a higher prevalence of high-risk nomophobia. Key predictors, including younger age, greater daily screen time, and elevated FoMO, were consistently associated with higher-risk profiles. These findings illustrate the critical role of targeted intervention efforts tailored to specific nomophobia patterns and student developmental stages. Moreover, the observed associations between nomophobia and social media use underscore the need to address digital behaviors within broader mental health frameworks. While the cross-sectional design limits causal inferences, this study lays a foundation for future longitudinal and cross-cultural research aimed at capturing the developmental trajectories and contextual influences of nomophobia.

## Figures and Tables

**Table 1:** The descriptive statistics for the two groups.

Variables	High School Students	College Students
Total	446	667
Gender (% female)	269 (60.3%)	463 (69.4%)
Age (mean, SD)	16.18 (1.15)	19.84 (1.46)
Residence (% urban)	149 (33.4%)	218 (32.7%)
Hours of smartphone use (mean)	0.55 per day	6.70 per day

**Table 2:** The group difference in nomophobia and its four dimensions.

	High School Students	College Students	*t*	Cohen’s d
Variables	M (*SD*)	M (*SD*)		
1. Nomophobia	3.64 (1.38)	4.01 (1.31)	−4.48 ***	0.27
2. Not being able to access information	3.62 (1.43)	3.88 (1.38)	−3.05 **	0.18
3. Giving up convenience	3.76 (1.63)	4.01 (1.49)	−2.60 **	0.16
4. Losing connectedness	4.02 (1.71)	4.49 (1.55)	−4.61 ***	0.29
5. Not being able to communicate	3.18 (1.67)	3.67 (1.62)	−4.87 ***	0.30

Note. ** *p* < 0.01, *** *p* < 0.001.

**Table 3:** Results of measurement invariance for nomophobia questionnaire.

Model	MLR χ^2^	df	CFI	TLI	SRMR	RMSEA	Model Comparison	ΔCFI	ΔRMSEA
Across gender									
(A) Configural	728.53	196	0.930	0.914	0.046	0.070			
(B) Metric invariance	744.21	208	0.929	0.918	0.046	0.068	B vs. A	–0.001	–0.002
(C) Scalar invariance	786.07	220	0.925	0.918	0.047	0.068	C vs. B	–0.004	–0.000
Across educational stage									
(A) Configural	768.26	196	0.924	0.908	0.047	0.073			
(B) Metric invariance	829.91	208	0.918	0.905	0.054	0.073	B vs. A	–0.006	0.000
(C) Scalar invariance	971.72	220	0.901	0.892	0.062	0.079	C vs. B	–0.017	0.006
(D) Partial scalar invariance	898.65	214	0.910	0.899	0.058	0.076	D vs. B	–0.008	0.003

Note: MLR = maximum likelihood robust.

**Table 4:** Nomophobia profile solutions for both high school and college students.

	AIC	BIC	aBIC	Entropy	BLRT(*p*)	LMRT(*p*)	Profile Prevalence
High school students							
2-Profile	26,152.20	26,353.12	26,197.62	0.937	<0.001	<0.001	0.503/0.497
3-Profile	24,964.91	25,235.53	25,026.07	0.951	<0.001	0.001	0.515/0.288/0.197
4-Profile	24,560.19	24,900.51	24,637.11	0.943	<0.001	0.351	0.398/0.267/0.188/0.147
College students							
2-Profile	38,511.39	38,731.59	38,576.01	0.938	<0.001	<0.001	0.614/0.386
3-Profile	37,131.88	37,428.46	37,218.91	0.917	<0.001	0.002	0.459/0.305/0.236
4-Profile	36,667.68	37,040.66	36,777.13	0.923	<0.001	0.153	0.450/0.247/0.156/0.148

**Table 5:** Model fit indices for multi-group profile similarity models for nomophobia.

Model	*#fp*	LL	AIC	BIC	aBIC	Entropy
1. Configural similarity	133	−31,650.63	63,567.26	64,233.51	63,811.07	0.957
2. Structural similarity	85	−31,774.63	63,719.26	64,145.06	63,875.08	0.956
3. Dispersion similarity	69	−31,789.86	63,717.73	64,063.38	63,844.22	0.956
4. Distributional similarity	67	−31,800.46	63,734.92	64,070.55	63,857.74	0.956

Note: *#fp* = number of free parameters; LL = loglikelihood.

**Table 6:** Multinomial logistic regression results.

		High vs. Low Nomophobia		Moderate vs. Low Nomophobia	
		*B*	OR	*B*	OR
Gender	College students	0.41	1.51	0.34	1.41
	High school students	−0.09	0.95	−0.05	0.91
Age	College students	−0.11	0.90	0.01	1.01
	High school students	−0.39 **	0.67 ***	−0.15	0.86
Residence	College students	−0.32	0.73	−0.12	0.88
	High school students	−0.22	0.80	−0.11	0.89
DPST	College students	0.05	1.06	0.02	1.02
	High school students	0.14 ***	1.15 **	0.11 **	1.12 **
FoMO	College students	2.53 ***	12.60 ***	1.53 ***	4.61 ***
	High school students	2.63 ***	13.92 ***	1.30 ***	3.68 ***

Note. *N* = 680, ** *p* < 0.01, *** *p* < 0.001, OR = odds ratio, reference group = low nomophobia profile, FoMO = fear of missing out, DPST = daily phone screen time.

**Table 7:** Chi-square analysis results for differences across three nomophobia profiles.

		High Nomophobia Profile	Moderate Nomophobia Profile	Low Nomophobia Profile	Overall χ^2^ (df)	Effect Size (Cramér’s *V*)
		M (SE)	M (SE)	M (SE)		
ASNSU	College students	2.68 (0.06)	2.49 (0.04)	2.16 (0.05)	χ^2^(2) = 49.68 ***	0.19
	High school students	2.76 (0.11)	2.33 (0.05)	1.76 (0.05)	χ^2^(2) = 90.26 ***	0.32
PSNSU	College students	3.56 (0.06)	3.29 (0.02)	2.85 (0.02)	χ^2^(2) = 58.55 ***	0.21
	High school students	4.01 (0.09)	3.14 (0.06)	2.63 (0.08)	χ^2^(2) = 133.82 ***	0.39

Note. *** *p* < 0.001, ASNSU = active social network site use, PSNSU = passive social network site use.

## Data Availability

The study data and materials can be made available on reasonable request from the corresponding author due to restrictions (privacy and ethical reasons).
